# Differences in Tissue Distribution of Cyano–B12 and Hydroxo–B12 One Week after Oral Intake: An Experimental Study in Male Wistar Rats

**DOI:** 10.3390/nu10101487

**Published:** 2018-10-12

**Authors:** Eva Greibe, Ole Nymark, Sergey N. Fedosov, Christian W. Heegaard, Ebba Nexo

**Affiliations:** 1 Department of Clinical Biochemistry, Aarhus University Hospital, Palle Juul-Jensens Boulevard 99, 8200 Aarhus N, Denmark; au335591@post.au.dk (O.N.); ebbanexo@rm.dk (E.N.); 2 Department of Molecular Biology and Genetics, Aarhus University, Gustav Wieds Vej 10, 8000 Aarhus, Denmark; SNFedosov1960@gmail.com (S.N.F.); cwh@mbg.au.dk (C.W.H.)

**Keywords:** cobalamin, vitamin B12, hydroxo–B12, cyano–B12, tissue distribution, rats

## Abstract

Foods contain natural vitamin B12 forms, such as hydroxo–B12 (HO–B12), whereas vitamin pills contain the synthetic cyano–B12 (CN–B12). Recent studies in rats showed different tissue distributions of CN–B12 and HO–B12 24 h after oral administration. Here, we investigate whether these differences are sustained or leveled out with time in both B12-deplete and -replete rats, thereby assessing if the two forms are equally good at maintaining a normal B12 status. Male Wistar rats were fed diets with low (*n* = 16) or high (*n* = 12) B12 content for 17 days. At day 10, the rats received a single oral dose of [^57^Co]-labeled CN–B12 or HO–B12 (*n* = 6 and *n* = 8, respectively, in each diet group). The rats were sacrificed on day 17 and endogenous B12 and [^57^Co]–B12 were measured in liver, kidney, and plasma. We found that the low-B12 diet introduced a B12-deplete state as judged from medians of endogenous B12 compared to rats on a (high-B12 diet): Plasma (565 (1410) pmol/L), liver (28.2 (33.2) pmol/g), and kidneys (123 (1300) pmol/g). One week after oral administration, the labeled B12 was distributed as follows: HO–B12 > CN–B12 (liver) and CN–B12 > HO–B12 (kidneys, plasma). The tissue/plasma ratios showed different equilibriums for labeled CN–B12 and HO–B12 in the B12-deplete and -replete groups. The equilibrium of endogenous B12 resembled [^57^Co]CN–B12 in replete rats but differed from both [^57^Co]CN–B12 and [^57^Co]HO–B12 in deplete rats. The data suggest long-term differences in tissue utilization of the two B12 forms and warrant further studies concerning the possible benefits of consuming HO–B12 instead of CN–B12 in oral B12 replacement.

## 1. Introduction

Vitamin B12 (B12, cobalamin) is an essential nutrient commonly obtained as hydroxo–B12 (HO–B12), 5′-deoxyadenosyl–B12 (Ado–B12), and methyl–B12 (CH_3_–B12), naturally found in foods of animal origin, or alternatively as the synthetic form, cyano–B12 (CN–B12), used in fortified foods and vitamin pills [1]. As Ado–B12 and CH_3_–B12 are converted into HO–B12 upon exposure to UV light [2], HO–B12 is a natural and abundant form of B12 in the diet [1]. 

Inadequate intake or impaired intestinal absorption of B12 can lead to a deficiency where the tissue stores of the vitamin become depleted. In humans, deficiency is associated with severe neurological and hematological symptoms and is treated with B12 injections or oral supplementation, depending on the cause and its severity [3].

Several studies in humans and rats suggest that CN–B12 and HO–B12 are absorbed equally well from the diet [4,5,6,7,8,9]. However, recent laboratory experiments revealed a discrepancy in the post-absorptive metabolism between the two B12 forms in rats. We found that acute oral administration of HO–B12 caused a relatively high liver accumulation, whereas supplementation with CN–B12 led to an increased accumulation in kidneys, brain, and plasma at 24 h [7,8]. The dissimilarity in accumulation of HO–B12 and CN–B12 prompted us to investigate if these short-term differences level out with time or reflect a sustained difference between the two B12 forms. For this purpose, we investigated the tissue accumulation of labeled CN–B12 and HO–B12 one week after oral administration in B12-deplete and -replete rats.

## 2. Materials and Methods

### 2.1. Animals

Male Wistar rats (RjHan:WI) (*n* = 28) from Janvier Labs, France, were used in the experiment. This strain of rats was chosen as it is commonly used for animal studies in B12 research [8,10,11]. The study was authorized by the Danish Animal Experimental Inspectorate, in agreement with the EU directive 2010/63/EU on animal experiments (approval number: 2016-15-0201-00984) and was conducted in agreement with the ARRIVE guidelines. The study was performed at the animal facility at the Health Faculty, Aarhus University, Denmark, and the institutional and national guidelines for care and use of animals were strictly followed. The rats were checked daily for any health or welfare problems, such as signs of pain, suffering, or distress, and all efforts were made to minimize suffering.

The rats were housed in pairs in standard cages (Makrolon 1291 H type III H, 800 cm^2^, Techniplast, Buguggiate, Italy) in a controlled environment (20.0 ± 0.5 °C; 60% humidity), with a 12 h light–dark cycle. Bedding material (asp chips, Tapvei, Kortteinen, Finland) and soft paper wool (LBS biotech, Surrey, United Kingdom) was changed frequently.

The rats were allowed two weeks of acclimatization in the animal facility before the experiment was initiated. During this time, the rats received a standard stock rat fodder (Altromin 1324, Brogaarden, Lynge, Denmark) containing 24 µg/kg of B12 (according to the manufacturer) and had free access to tap water. During the study, the rats received special diets with either low or high B12 content (see study design in Section 2.2). In practice, the study on the low-B12 diet (November 2016) was conducted three months before the study on the high-B12 diet (February 2017), and the two groups of rats had different median ages at the time of sacrifice (B12-deplete rats, 8 weeks; B12-replete rats, 12 weeks; see Discussion concerning a possible impact). Aside from this, there was no major difference in the experimental setups between the two sub-studies.

### 2.2. Study Design

**Figure 1:**
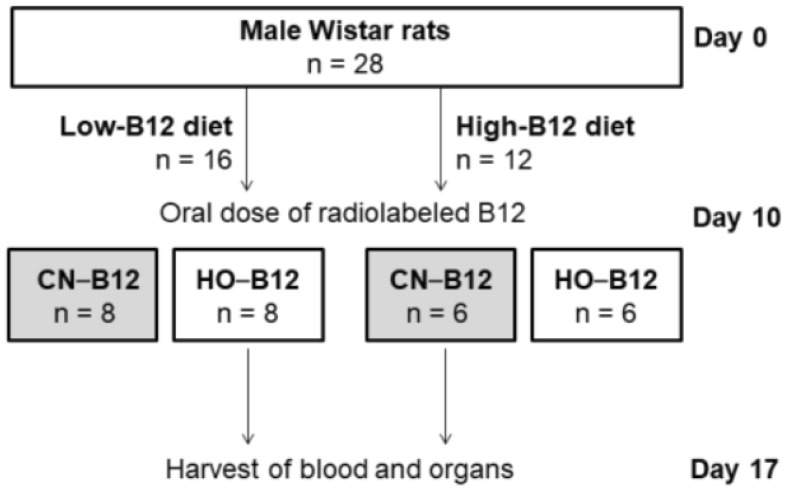
Study design. Abbreviations: CN–B12: [^57^Co] cyano–B12 (CN–B12); HO–B12: [^57^Co] hydroxo–B12 (HO–B12).

The design is outlined in Figure 1. Rats (*n* = 28) received diets with either low (*n* = 16) or high (*n* = 12) B12 content (see Section 2.3 for information on diets) for 10 days. At this point (day 10), each group was divided further into two subgroups and given an oral dose of radioactive labeled B12, either [^57^Co]CN–B12 or [^57^Co]HO–B12 (see Section 2.4 on oral administrations). The labeled B12 deviates from the unlabeled B12 only by having a central Co^57^ isotope instead of Co^59^, and the label remains within the organic cage of B12 for its lifetime. Both radioactive and nonradioactive B12 are metabolized identically. The oral administration was done by gastric gavage employing a 20-gauge needle. The rationale for using this technique was to ensure that all rats received the same exact amount of labeled B12. One week after oral administration (day 17), a blood sample was taken by puncture of the sublingual vein with a 23-gauge needle, allowing blood to run from the mouth of the rat and directly into a 4 mL lithium heparin tube (BD Vacutainer). The blood samples were centrifuged at room temperature for 9 min at 1850 g, whereupon plasma was collected and stored at −20 °C until analysis. After this, the rats were anesthetized with isoflurane gas and sacrificed by cervical dislocation. Liver and kidneys were collected, weighed, and snap-frozen in liquid nitrogen before being stored at −80 °C until further processing (see Section 2.5 and Section 2.6).

### 2.3. Diets with Low and High B12 Content

All rats had free access to food and water throughout the study. The diets with low B12 content (Altromin C-1024, indicated to contain <11 µg/kg of B12) and high B12 content (Altromin 1324, indicated to contain 24 µg/kg of B12) were purchased from Brogaarden, Lynge, Denmark. The high-B12 diet is the standard diet in the animal facility, and all rats received it during the two-week acclimatization period in the animal facility before the study was initiated. In-house analyses, as previously described [12], were used to determine the form of B12 in the diets. 

### 2.4. Solutions for Oral Administration

On day 10 (see Figure 1), the rats received an oral dose of 1 pmol radioactive labeled B12 (approx. 180,000 cpm) as either [^57^Co]CN–B12 or [^57^Co]HO–B12 in 0.5 mL water. Commercially available [^57^Co]CN–B12 (1.75 µCi/mLand 0.41 µCi/pmol CN–B12) (MP Biomedicals, Solon, Ohio, USA, catalogue no. 06B-430000) was used. [^57^Co]HO–B12 was made from [^57^Co]CN–B12, as previously described [7]. The labeled B12 was adjusted to the desired concentration by addition of unlabeled CN–B12 (cyanocobalamin, Sigma–Aldrich, Brøndbyvester, Denmark) or HO–B12 (Vibeden, Sandoz, Denmark). The exact amount of radioactivity (in cpm) administered to each group was measured by gamma counting of a test aliquot (0.5 mL). To reduce the bias from potential inaccuracies in the oral administration procedure, the test aliquot was collected by imbibing a 0.5 mL solution with the gastric gavage needle in the same manner as for oral administration in a rat and then transferring the content into a test tube. The term [^57^Co]B12 will be used throughout the text to cover both forms of the labeled B12 ([^57^Co]CN–B12 and [^57^Co]HO–B12).

### 2.5. Quantification of Endogenous B12 in Plasma and Tissues

Endogenous plasma B12 was measured directly on the Advia Centaur CP Immunoassay System (Siemens), either undiluted (B12-deplete rats) or using a 1:2 dilution with 0.9% NaCl (B12-replete rats). The tissues were thawed on ice and endogenous tissue B12 was extracted from the liver and kidneys by homogenizing 0.25 g of tissue in 750 mLof Na–acetate buffer (0.4 mol/L, pH 4.4) using the Precellys 24 (Bertin Technologies, Montigny-le-Bretonneux, France) with three centrifugation cycles of 20 s at 6,800 rpm, with 30 s pauses between cycles. After homogenization, 20 µL of potassium cyanide (KCN)solution (30 mmol/L) was added to convert all B12 in the samples to CN–B12. The KCN solution was made by dissolving 100 mg KCN (Sigma–Aldrich 60178-25 g, Germany) in 50 mL water. Then, the mixtures were boiled for 10 min and centrifuged for 40 min at 20,000× *g* and 4 °C, and the supernatants were collected and stored at −20 °C until analyzed. The supernatants were measured for total B12 content on the Advia Centaur CP Immunoassay System (Siemens) after dilution with 0.9% solution of NaCl. Supernatants from the B12-deplete rats were diluted 1:10 (liver) and 1:50 (kidneys). Supernatants from the B12-replete rats were diluted 1:10 (liver) and 1:500 (kidneys). The dilutions were chosen to ensure that the B12 concentrations would be within the measurements range (100-1476 pmol/L) of the Advia Centaur CP Immunoassay system. The results were expressed as pmol B12 per gram of a wet tissue.

### 2.6. Quantification of [^57^Co]B12 Accumulation in Plasma and Tissues

[^57^Co]B12 was measured by gamma counting using the Wizard Automatic Gamma Counter (Perkin Elmer). In practice, all tissues were thawed on ice, cut into smaller pieces, and transferred to tubes for the gamma counter. All tubes were counted to obtain the whole-organ cpm. Results are expressed as the fraction (%) of the total administered dose (cpm) of [^57^Co]B12 per animal.

### 2.7. Statistical Methods

The number of animals per group was based on power calculations using a multiple linear regression model, showing a statistical power of 80% and a confidence level of 95%. The calculations were based on an earlier study [8], where a two-week low-B12 diet caused an 85% decrease in mean plasma B12 (1330 pmol/L to 190 pmol/L). Based on this study, we anticipated a mean plasma B12 decrease of 60% for rats fed a similar low-B12 diet for 10 days. Sample size calculations suggested *n* = 5 in each subgroup ([^57^Co]CN–B12 and [^57^Co]HO–B12). In order to account for possible outliers and sick animals, we included eight rats in each subgroup on a low-B12 diet (*n* = 16 in total) and six rats in each subgroup on a high-B12 diet (*n* = 12 in total). 

The D’Agostino–Pearson omnibus test was used to determine whether the data followed the Gaussian distribution, and logarithmic transformation was used to obtain normal distribution. Differences between the two groups were determined using the unpaired t-test. *p-*values of ≤0.05 were accepted as statistically significant. The data analysis was performed using the statistical software available in GraphPad Prism version 7.03 (GraphPad, La Jolla, CA, USA).

## 3. Results

We present data on the tissue distribution of radiolabeled CN–B12 and HO–B12 one week after oral administration in B12-deplete (low-B12 diet) and B12-replete (high-B12 diet) rats. The design is depicted in Figure 1. The four weeks younger B12-deplete rats weighed less (median (range)) (303 (274–350) g) than the B12-replete rats (397 (383–414) g) (*p* < 0.0001) (see Discussion), and the same pattern was seen for the kidney weights (B12-deplete: 2.6 (2.3–3.0) g; B12-replete: 3.1 (2.7–3.4) g) (*p* < 0.0001). However, no difference in the liver weights was found between the two groups (B12-deplete: 15.1 (12.1–20.3) g; B12-replete: 14.4 (13.1–16.5) g) (*p* = 0.23).

### 3.1. B12 Content and Forms in the Diet

We measured the form of B12 in the diets. The low-B12 diet (<11 µg/kg of B12) contained mainly HO–B12 (~70%, apparently from the food itself) and the high-B12 diet (24 µg/kg of B12) contained mainly CN–B12 (~70%, apparently from the additives). All animals thrived throughout the study, revealing no symptoms related to differences in B12 intake.

### 3.2. Endogenous B12 Distribution in B12-Deplete and B12-Replete Rats

The acute oral dose of 1 pmol [^57^Co]B12 given to all animals was too small to influence the endogenous B12 levels, even in the B12-deplete rats. After 17 days on the diets with low or high B12, plasma and tissues were harvested from the animals. The results are reported as pmol B12/g of wet tissue (liver and kidneys) and pmol B12/L (plasma) (Table 1), and this normalization is expected to compensate for the different weight of organs. Consistent with findings in other rat studies, the B12-replete rats showed higher concentrations of endogenous B12 in plasma and tissues compared with the B12-deplete rats [8,12].

### 3.3. Distribution of [^57^Co]B12 One Week after Oral Administration to B12-Deplete and B12-Replete Rats

**Figure 2:**
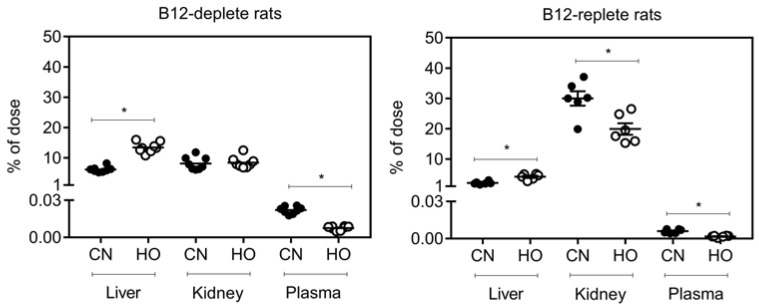
Tissue [^57^Co]B12 accumulation in B12-deplete and -replete rats one week after oral administration of [^57^Co]CN–B12 or [^57^Co]HO–B12. Results are given as the percentage of cpm of the given dose of [^57^Co]B12 in liver and kidney (whole organs) and in plasma (per mL). [^57^Co]CN–B12 administration is shown with filled black symbols (*n* = 8 for B12-deplete rats, *n* = 6 for B12-replete rats). [^57^Co]HO–B12 administration is shown with open white symbols (*n* = 8 for B12-deplete rats, *n =* 6 for B12-replete rats). Horizontal lines show mean values. Scatter symbols show the values for each individual rats. Differences between administrations of [^57^Co]CN–B12 and [^57^Co]HO–B12 were estimated with the unpaired *t*-test. *p*-values ≤ 0.05 were judged as statistically significant and are indicated by asterisks. Abbreviations: CN: [^57^Co]CN–B12; HO: [^57^Co]HO–B12.

We studied the distribution of [^57^Co]CN–B12 and [^57^Co]HO–B12 in liver, kidneys, and plasma one week after oral administration to B12-deplete and -replete rats. The results are shown in Figure 2 as the fractions of administered [^57^Co]B12 accumulated in each organ after one week. In the B12-deplete rats, we recovered the following median values of the administered dose (liver + kidney): 14% of [^57^Co]CN–B12 and 22% of [^57^Co]HO–B12. This posed a highly significant difference between the two B12 forms (*p* < 0.0001). In the B12-replete rats, these figures were 32% ([^57^Co]CN–B12) and 21% ([^57^Co]HO–B12) (*p* = 0.03). Interestingly, the total recovered dose of [^57^Co]CN–B12 (liver + kidney) varied greatly among B12-deplete (14%) and -replete (32%) animals (*p* < 0.0001), whereas no difference in the recovered [^57^Co]HO–B12 was found between B12-deplete (22%) and -replete (21%) animals (*p* > 0.05). The first observation was explained by a preferential accumulation of CN–B12 in the kidneys of the B12-replete rats (with larger organs) that was greatly suppressed in the kidneys of the B12-deplete animals (with smaller organs). In general, we found the distributional differences between the tissues to depend on both B12 status and the [^57^Co]B12 form administered. For both groups of rats, more [^57^Co]HO–B12 accumulated in the liver and less in plasma compared with [^57^Co]CN–B12, suggesting that the two B12 vitamers deposit differently in the tissues. Furthermore, the B12-replete rats had a higher accumulation of [^57^Co]CN–B12 than [^57^Co]HO–B12 in the kidneys, but this difference was not observed for the B12-deplete rats. These findings are in accord with the distributional differences previously observed 24 h after oral administration of the two forms of B12 [7,8].

**Figure 3:**
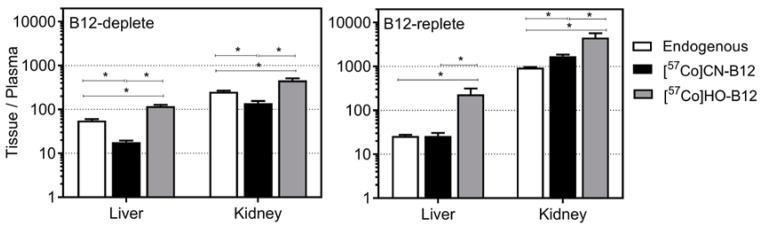
Ratios of endogenous B12 and [^57^Co]B12 in kidney and liver. Ratios (mean tissue B12/mean plasma B12) of endogenous B12 (pmol/g)/(pmol/ml) (white bars, *n* = 16 B12-deplete, *n* = 12 B12-replete) and [^57^Co]B12 (cpm/g)/(cpm/mL) accumulated one week after administration of oral [^57^Co]CN–B12 (black bars, *n* = 8 B12-deplete, *n* = 6 B12-replete) and [^57^Co]HO–B12 (grey bars, *n* = 8 B12-deplete, *n* = 6 B12-replete) in rats kept on a diet with low-B12 or high-B12 for 17 days. The ratios (bars) are shown as mean ± SEM. X-axis indicates the tissues examined (liver and kidney). Y-axis indicates tissue/plasma ratio on a log-scale. Dashed horizontal lines are depicted to simplify comparison of bars. Differences between adjacent bars were determined by the unpaired *t*-test. *p*-values ≤ 0.05 were judged as statistically significant and are indicated by asterisks.

Figure 3 presents the tissue/plasma ratios for the [^57^Co]B12 concentrations one week after oral administration, where the tissue ↔ plasma exchange should be equilibrated. In B12-deplete rats, all ratios of endogenous B12 (Figure 3, open bars) were situated between [^57^Co]HO–B12 (Figure 3, grey bars) and [^57^Co]CN–B12 (Figure 3, black bars). This means that the two B12 forms behaved differently during tissue compartmentalization. In B12-replete rats, a considerable similarity was found between ratios of endogenous B12 and CN–B12. Collectively, the observations were interpreted in terms of different equilibrium characteristics of the two B12 forms, prevailing within the pool of endogenous B12; see Discussion for details.

Alignment of B12-deplete and –replete rats shows that such depletion significantly decreased the kidney/plasma B12 ratios for all B12 forms (endogenous, CN–B12, HO–B12), correlating with studies suggesting that the kidney is the main storage organ of B12 in rats [11,13,14]. By contrast, liver/plasma ratios showed upon depletion some increase for the endogenous and HO–B12 forms, but no change for CN–B12. In all cases, [^57^Co]HO–B12 gave rise to higher tissue/plasma ratios than [^57^Co]CN–B12, pointing to a better tissues compartmentalization.

## 4. Discussion

We investigated the tissue distribution of two forms of B12, CN–B12 and HO–B12, in B12-deplete and B12-replete rats. We found that the distributional differences between the tissues depend on both the B12 status of the rats and the form of B12 administered.

### 4.1. Introduction of B12 Depletion

We induced B12 depletion in 16 rats by keeping them on a low-B12 diet for 17 days. Endogenous B12 in plasma and tissues confirmed B12 depletion, which was mostly pronounced in the kidneys, a known storage organ of B12 in the rat [11,13,14], and to a much lesser extent in the liver. These findings are in agreement with previous rat studies [8,11,13] and confirm the conjecture that kidneys accumulate pools of free B12 during sufficient B12 supply [11], but recirculate the vitamin for its utilization by the rest of the body under B12 deprivation [11,13]. In this way, the amounts of enzyme-bound B12 coenzymes in the liver and other key organs are preserved so that the activities of the B12-dependent enzymes, methylmalonyl–CoA mutase and methionine synthase, are reduced as little as possible during B12 depletion [13].

### 4.2. Distribution of Labeled CN–B12 and HO–B12

When supplying [^57^Co]CN–B12 and [^57^Co]HO–B12 to B12-deplete and B12-replete rats, we expected that the short-term differences in the tissue distribution of the two B12 forms would level out after one week. Yet, we still found different distributions resembling those at 24 h [8]. The liver accumulated more [^57^Co]HO–B12 than [^57^Co]CN–B12, whereas the opposite picture was seen in the kidneys (only in B12-replete rats) and plasma. A recent rat study examined the endogenous B12 tissue distribution after two weeks of dietary intake of CN–B12 and HO–B12 [12]. It was judged that HO–B12 appears to be a better source of the active coenzymes, Ado–B12 and CH_3_-B12, than CN–B12, in spite of a lower B12 plasma concentration [12]. This could be at least partly explained by a slower conversion of CN–B12 to coenzymes compared to that of HO–B12 [15,16]. Clinical studies also showed that administration of CN–B12 creates high amounts of the circulating unconverted vitamin [17], suggesting the same pattern in humans. Our findings of persistent differences in the compartmentalization of CN–B12 and HO–B12 after acute oral administration support the data about a disparity of the endogenous B12 forms [12]. However, further studies are needed to clarify whether the supply of HO–B12 provides more tissue coenzymes if compared with CN–B12, and whether these observations are of any relevance in a clinical setting. In this context, it would also be of interest to include the two coenzyme forms, CH_3_–B12 and Ado–B12, since both of them are gaining popularity as alternatives to CN–B12 for oral supplementation in humans [18].

### 4.3. Tissues/Plasma Ratios for CN–B12 and HO–B12

We also explored the tissue/plasma ratios in order to gain knowledge on the steady-state dynamics of CN–B12 and HO–B12. We speculated with sufficient confidence that the acute [^57^Co]B12 doses were equilibrated between different compartments within one week after administration. Therefore, the deviating tissue/plasma ratios for CN–B12 and HO–B12 suggest different equilibriums for the two B12 forms. The most puzzling observation concerned the absent equality between endogenous B12 and any labeled form in the B12-deplete rats (Figure 3A), contrasted with a noticeable similarity between endogenous B12 and CN–B12 in the replete rats (Figure 3B). We speculate whether these observations were caused by different amounts of CN–B12 in the diets. The deplete rats received only minor quantities of CN–B12 and could not store it. In addition, the previously accumulated inert CN–B12 is expected to be converted to the catalytically active B12 forms (i.e., the coenzymes being in a steady state with HO–B12). This means that the tissue/plasma ratios of endogenous B12 can bear some resemblance to HO–B12, but would differ from CN–B12. The situation is different in the B12-replete rats. They receive high quantities of CN–B12 that accumulates in the tissues, shifting the equilibrium characteristics in favor of CN–B12. The important consequence of the latter observation is that high level of B12 in a tissue does not automatically equal high “active” B12. We find these differences in metabolism and tissue distribution of CN–B12 and HO–B12 to be both surprising and important, when choosing the right form of B12 for oral supplementation. 

### 4.4. Thoughts on the Isotope Dilution Technique

In the 1970s, the isotope dilution technique was used to assess the total B12 amount in the body [19]. This method assumes that an injection of labeled B12 will distribute throughout the human body, similarly to the endogenous B12, and reach equilibrium within 5–10 days. At this point, the total amount of endogenous B12 in the body can be calculated by examination of labeled B12 and endogenous B12 in a representative biopsy, for example, from the liver [19]. In this way, the isotope dilution technique relies on the assumption that all B12 forms are equally distributed in the body. Since this is not the case, we have to stress tha isotope dilution techniques cannot be used without a correction for different forms of B12.

### 4.5. Study Limitations

The work has some limitations. The B12-deplete rats (low-B12 diet) were four weeks younger than the B12-replete rats (high-B12 diet), and had a lower total body weight at the time of sacrifice. Nevertheless, no consistent variation in the tissue and plasma contents of B12 were found in rats at 2, 12, and 24 months [20]. We have chosen not to compare the two groups, however. Instead, our findings provide new knowledge on the differences in the one-week accumulation of CN–B12 and HO–B12. We did not measure the intracellular metabolites, methylmalonic acid and homocysteine, and therefore can only assess on tissue B12 accumulation, but not on the degree of metabolic B12 deficiency.

## 5. Conclusions

The tissue distributions of labeled CN–B12 and HO–B12 one week after oral administration show great resemblance to the tissue distribution seen after 24 h, suggesting that the short-time pattern serves as a good predictor of the “true” long-time steady-state distribution. It also shows that there is a consistent difference in the tissue distributions of CN–B12 and HO–B12. We find that the compartmentalization of B12 in rats depends on B12 status and the vitamin form administered. Our data highlights the need for clinical investigations of long-term treatment with high doses of CN–B12 and HO–B12 in B12-deficient individuals, with the aim of exploring any possible benefits of using one B12 form or the other in oral replacement.

## Figures and Tables

**Table 1:** Endogenous B12 in liver, kidneys, and plasma of rats kept on low (B12-deplete rats) and high (B12-replete rats) B12 diets for 17 days. Results are given as median with (range).

	B12-Deplete Rats (*n* = 16)	B12-Replete Rats (*n* = 12)
Liver (pmol/g)	28.2 (23.4–41.1)	33.2 (25.2–46.7)
Kidneys (pmol/g)	123 (104–188)	1300 (1133–1512)
Plasma (pmol/L)	565 (353–646)	1410 (1150–1630)
